# A positive feedback loop of SRSF9/USP22/ZEB1 promotes the progression of ovarian cancer

**DOI:** 10.1080/15384047.2024.2427415

**Published:** 2024-11-12

**Authors:** Jing Wang, Ming Hu, Jie Min, Xin Li

**Affiliations:** Department of Gynecology II, Renmin Hospital of Wuhan University, Wuhan, Hubei Province, P. R. China

**Keywords:** Ovarian cancer, SRSF9, USP22, ZEB1

## Abstract

Ovarian cancer (OC) is recognized as the most lethal type of gynecological malignancy, making treatment options challenging. Discovering novel therapeutic targets will benefit OC patients. This study aimed to reveal the mechanism by which SRSF9 regulates OC progression. Cell proliferation was determined via CCK-8 assays, whereas cell migration and invasion were monitored via Transwell assays. Western blotting and qPCR assays were used to detect protein and mRNA alterations. RNA pull-down, RNA immunoprecipitation (RIP), and actinomycin D experiments were performed to investigate the relationships between SRSF9 and USP22. Co-IP was used to validate the interaction between USP22 and ZEB1. Chromatin immunoprecipitation (ChIP) and dual-luciferase reporter assays were used to verify the regulatory effect of ZEB1 on the transcription of SRSF9. Subcutaneous xenograft models were established to evaluate the impact of SRSF9 on tumor development. Knockdown of SRSF9 significantly suppressed the proliferation, invasion, migration, tumorigenicity, and epithelial‒mesenchymal transition (EMT) of OC cells. SRSF9 can bind to USP22 mRNA, increasing its stability. Moreover, the overexpression of USP22 reversed the impact of SRSF9 silencing on malignant phenotypes. USP22 can mediate the deubiquitination of ZEB1, thereby enhancing the progression of OC. Furthermore, ZEB1 upregulated SRSF9 expression through transcriptional activation, thus establishing a positive feedback loop. SRSF9 enhanced the malignant characteristics of OC through a positive feedback loop of SRSF9/USP22/ZEB1. This functional circuit may help in the development of novel therapeutic approaches for treating OC.

## Introduction

Ovarian cancer (OC) is a prevalent cancer in women and ranks eleventh in terms of incidence and fifth in mortality worldwide.^1^ Annually, approximately 140,000 women die from OC, primarily due to late-stage diagnoses and a lack of effective treatments.^2,3^ The primary approach to treating OC involves cytoreductive surgery to eradicate visible tumors, followed by platinum-based chemotherapy, especially for patients with high-grade serous OC.^4^ Unfortunately, despite approximately 80% of OC patients receiving this standard treatment regimen, around 70% experience relapse with resistance to standard chemotherapy.^5^ Research into molecular alterations in OC patient populations has revealed promising prospects for identifying novel diagnostic and therapeutic targets to improve outcomes for individuals battling this disease.

RNA-binding proteins (RBPs), a group of proteins capable of interacting with RNA sequences both within and outside specific RNA regions, play critical roles in modulating the destiny and functionality of RNA.^6^ Several studies have indicated that RBPs are abnormally expressed in OC tissues and are involved in regulating OC progression.^7–9^ Serine/arginine-rich splicing factor 9 (SRSF9), which is considered a new biomarker and therapeutic target in various cancers, including colorectal cancer,^10^ hepatocellular carcinoma,^11^ and cervical cancer,^12^ plays a vital role in tumorigenesis. In addition, SRSF9 is overexpressed in OC tumor tissues, and high SRSF9 levels are associated with a poor prognosis in OC patients.^13^ However, the exact involvement of SRSF9 in OC has yet to be investigated despite these results.

Zinc finger E-box-binding homeobox 1 (ZEB1) is predominantly recognized as a transcriptional regulator that performs protumor functions in several malignant diseases. By suppressing the expression of E-cadherin, a critical transmembrane protein essential for maintaining epithelial characteristics, ZEB1 drives the initiation of epithelial‒mesenchymal transition (EMT). Importantly, elevated ZEB1 levels have been linked to advanced disease stages, metastasis, and adverse prognostic outcomes in OC patients.^14,15^ Research has shown that high ZEB1 expression in tumors may be related to decreased ZEB1 ubiquitination. Ubiquitin‑specific protease 51 (USP51) regulates drug resistance in lung cancer by mediating ZEB1 deubiquitination.^16^ USP10 inhibits ZEB1-mediated colorectal cancer metastasis by regulating ZEB1 ubiquitination and protein stability.^17^ In liver cancer cells, USP22 regulates ZEB1 ubiquitination to influence ZEB1-mediated gene transcription and promote tumor progression.^18^ USP22 has been shown to have procancer effects in various tumors, and knocking down USP22 can inhibit OC cell proliferation.^19^ However, further investigations are needed to determine whether USP22 mediates ZEB1 ubiquitination and participates in tumor progression in OC.

Furthermore, we analyzed potential candidate mRNAs that can be bound by SRSF9 using the ENCORI dataset (https://rnasysu.com/encori/) and found that SRSF9 could bind to USP22 mRNA. Moreover, we predicted that ZEB1 could be a potential transcription factor of SRSF9 via the JASPAR dataset (https://jaspar.genereg.net/). Therefore, we hypothesized that an interaction may exist among SRSF9, USP22, and ZEB1, and the objective of this study was to explore the role of SRSF9 in the pathogenesis of OC and to elucidate the mechanism of the interactions among SRSF9, USP22, and ZEB1.

## Methods

### Cell culture

The human OC cell lines A2780 and SKOV3 were obtained from the American Type Culture Collection (Manassas, VA, USA). These cells were cultured in Dulbecco’s modified Eagle’s medium (Gibco, Grand Island, NY, USA) supplemented with 10% fetal bovine serum under standard conditions at 37°C and 5% CO_2_ in a humidified incubator.

### Cell transfection

For the knockdown of SRSF9 and USP22, shRNAs targeting SRSF9 (sh-SRSF9) and USP22 (sh-USP22) and a negative control (sh-NC) were synthesized by GeneChem (Shanghai, China). For the overexpression of USP22 and ZEB1, the USP22 or ZEB1 sequence was inserted into the pcDNA3.1 vector (oe-USP22, oe-ZEB1; RiboBio, Guangzhou, China), and an unedited pcDNA3.1 vector was utilized as the negative control (oe-NC) in the experiments. A2780 and SKOV3 cell lines were transfected with the aforementioned plasmids using Lipofectamine 3000 (Invitrogen, Carlsbad, CA, USA) for 2 days. The transfection efficiency was assessed prior to the designated experimental procedures.

### Cell counting kit-8 (CCK-8) assay

Cell viability was determined by a CCK-8 assay. Briefly, A2780 and SKOV3 cells transfected with their respective vectors were seeded in a 96-well plate (5 × 10^3^ cells per well). At 0, 24, 48, and 72 hours, 10 μL of CCK-8 solution (TransGen, Beijing, China) was added to each well, and the cells were then incubated for an additional 4 hours. The absorbance at 450 nm was subsequently determined using a microplate reader.

### Transwell assay

Serum-free medium (200 μL) containing approximately 1 × 10^5^ OC cells subjected to various treatments was plated into the upper chamber (Corning, New York, USA) with or without precoated Matrigel (BD Biosciences, San Jose, California, USA) to study invasion or migration, while 600 μL of medium supplemented with 10% FBS was added to the lower chambers. After a 48-hour incubation period, the cells in the lower chambers were fixed with 4% formaldehyde and then stained with 0.1% crystal violet (Solarbio, Beijing, China) for further analysis. The quantification of invasive or migrated cells was performed using a light microscope from Olympus, headquartered in Tokyo, Japan.

### Quantitative real-time PCR (qPCR) assay

Cellular RNA was isolated using TRIzol Reagent (Invitrogen) and then utilized for cDNA synthesis with the SuperScript^TM^ IV First-Strand Synthesis System (Invitrogen). The expression levels of SRSF9, USP22, and ZEB1 were quantified via qPCR with SYBR Green PCR Master Mix (Takara, Tokyo, Japan) and analyzed with the 2^−ΔΔCt^ method. GAPDH was used as an internal control, and the primers utilized in the qPCR assay are listed in Table 1.

**Table 1.** The primer sequences used in this study.

Gene	Primer sequences
SRSF9	Forward: 5’-CCT GCG TAAACTGGATGACACC-3’
	Reverse: 5’-CCTGCTTTGGTATGGAGAGTCAC-3’
USP22	Forward: 5’-CTACCAGGAGTCCACAAAGCAG-3’
	Reverse: 5’-CACATACGTGGTGATCTTCCGC-3’
ZEB1	Forward: 5’-GGCATACACCTACTCAACTACGG-3’
	Reverse: 5’-TGGGCGGTGTAGAATCAGAGTC-3’
GAPDH	Forward: 5’-GTCTCCTCTGACTTCAACAGCG-3’
	Reverse: 5’-ACCACCCTGTTGCTGTAGCCAA-3’

### Western blot analysis

Total protein was extracted from OC cells or tumor tissues with RIPA lysis buffer (TransGen). The protein concentrations of the samples were detected using a BCA protein assay kit (Solarbio). Protein samples (40 μg) were subsequently separated on a 10% SDS polyacrylamide gel and electrotransferred onto a PVDF membrane (Millipore, Billerica, MA, USA). The blots were subsequently blocked in 5% skim milk and probed with primary antibodies, including anti-SRSF9 (ab236414, 1:2000, Abcam, Cambridge, MA, USA), anti-USP22 (ab195289, 1:2000, Abcam), anti-ZEB1 (ab203829, 1:500, Abcam), anti-E-cadherin (ab40772, 1:5000, Abcam), anti-N-cadherin (ab76011, 1:5000, Abcam), anti-Vimentin (ab92547, 1:2000, Abcam), and anti-β-actin (ab115777, 1:200, Abcam), overnight at 4°C. After washing, the membrane was incubated with appropriate secondary antibodies, followed by protein band detection using an ECL system (Thermo Fisher Scientific, Waltham, MA, USA).

### RNA pull-down assay

A Magnetic RNA‒Protein Pull-Down Kit (Thermo Fisher Scientific) was used for the RNA pull-down assay. Initially, the cells were lysed with Pierce IP Lysis Buffer for 5 minutes, followed by centrifugation at 13,000 ×*g* for 10 minutes. The lysates were collected and then coincubated with streptavidin magnetic beads conjugated with biotinylated sense or antisense USP22 (Sangon Biotech, Shanghai, China). The proteins bound to the RNA‒protein complexes were subsequently released from the magnetic beads by boiling for 10 minutes, and SRSF9 protein expression was evaluated via western blotting.

### RNA immunoprecipitation (RIP) assay

The RIP assay was conducted using the Magna RIP Kit (Millipore). A2780 and SKOV3 cells were washed with prechilled PBS and lysed in RIP buffer at 4°C for 30 minutes. Magnetic beads bound to human anti-SRSF9 antibodies (sc-293314, Santa Cruz Biotechnology, Santa Cruz, CA, USA) or normal rabbit IgG (30000–0-AP, Proteintech, Wuhan, China) were utilized to capture the RNAs for subsequent qPCR analysis to determine the expression level of USP22.

### RNA stability analysis

Actinomycin D (Beyotime, Shanghai, China) was administered as a treatment to assess RNA stability. OC cells were exposed to actinomycin D at a concentration of 5 μg/mL for 0, 4, or 8 hours, after which RNA was extracted. Subsequent qPCR analysis was performed to detect the mRNA level of ZEB1.

### Coimmunoprecipitation (Co-IP) assay

A co-IP assay was conducted to explore the interaction between USP22 and ZEB1. The cells were first washed with PBS and then lysed in 1.2 mL of lysis buffer for 30 minutes. The lysates were subsequently centrifuged at 14,000 rpm for 15 minutes at 4°C, after which the resulting supernatant was carefully transferred to a new column. The samples were divided into three parts: one for the input protein, and the other two were incubated with either anti-IgG (30000–0-AP, Proteintech) as a control or anti-USP22 (55110–1-AP, Proteintech) overnight at 4°C with gentle shaking. Protein G magnetic beads (Millipore) were subsequently added to the samples, which were subsequently incubated at 4°C for 6 hours. After immunoprecipitation, the samples were washed with lysis buffer and then subjected to three rounds of washing. The retained proteins were eluted by mixing with 30 μL of loading buffer at 100°C for 10 minutes. The protein complexes and input proteins were ultimately assessed via western blot analysis.

### Detection of ubiquitination

To analyze the ubiquitination level of ZEB1, an IP assay was conducted. Initially, OC cells were lysed and then centrifuged at 14,000 ×*g* for 15 minutes to collect the cell lysate. Following a 2-hour incubation with protein A/G magnetic beads (Thermo Fisher Scientific), the resulting supernatants were incubated at 4°C with a specific antibody targeting ZEB1 (21544–1-AP, Proteintech) overnight. Next, the beads were subjected to five rigorous washes with Western/IP lysis buffer, resuspended in loading buffer for SDS‒PAGE, and then subjected to western blot analysis with an anti-ubiquitin antibody (80992–1-RR; Proteintech).

### Chromatin immunoprecipitation (ChIP)

To determine the binding of ZEB1 to the SRSF9 promoter region, a ChIP assay was performed in OC cells using a commercial ChIP Kit (CST, Danvers, MA, USA) following the manufacturer’s instructions. Chromatin was immunoprecipitated with an anti-ZEB1 antibody (21544–1-AP, Proteintech) or control IgG (30000–0-AP, Proteintech) at 4°C for 12 hours. Finally, the resulting DNA fragments were amplified with specific primers targeting the ZEB1 binding site within the SRSF9 promoter.

### Dual-luciferase reporter assay

The ZEB1 binding sites within the SRSF9 promoter region and the fragment with mutated ZEB1 binding sites were integrated into the pmirGLO vector (Promega, Madison, WI, USA). In addition, oe-NC and oe-ZEB1 were cotransfected with the engineered luciferase reporter vector into cells. Following a 48-hour incubation period, the cell lysate was collected, and the firefly luciferase activity was determined by using a Dual-Luciferase Reporter Assay System (Promega). Renilla luciferase activity was used as the internal reference for normalization.

### Tumor implantation assay

The animal experiments were conducted following the guidelines of the institutional ethics committee (No. 20220508). Four-week-old female BALB/c nude mice were subcutaneously inoculated with A2780 cells (4 × 10^6^ cells) transfected with either sh-NC or sh-SRSF9. Tumor sizes were monitored weekly after cell injection, and on the 35th day postinjection, the mice were sacrificed. The tumor size and weight were measured, and slides of the tumor tissues were prepared.

### Immunohistochemistry (IHC) assay

For the IHC analysis of tumor tissues, the slides were deparaffinized and rehydrated using a gradient of alcohol solutions, followed by antigen retrieval. The slides were subsequently incubated for 60 minutes at room temperature in blocking solution containing 5% normal goat serum, 0.1% Triton X-100, and 3% H_2_O_2_ in PBS. The slides were then incubated overnight at 4°C with an anti-Ki-67 antibody (28074–1-AP, 1:1000, Proteintech). IHC staining was performed using horseradish peroxidase (HRP) conjugates, and the detection was carried out with DAB solution. Hoechst was used for nuclei counterstaining, and images were captured using an Olympus microscope.

### Statistical analysis

Each experimental procedure was repeated three times. The results are expressed as the mean ± standard deviation (SD). Pairwise comparisons between two groups were conducted using Student’s t test, whereas comparisons among three or more groups were performed via one-way analysis of variance (ANOVA) followed by Tukey’s post hoc test. GraphPad Prism 9.0 (GraphPad Software, La Jolla, CA, USA) was used for statistical analysis, where significance was defined as a *p* value less than 0.05.

## Results

### SRSF9 regulates cell proliferation, migration, invasion, and EMT in OC

**Figure 1.**
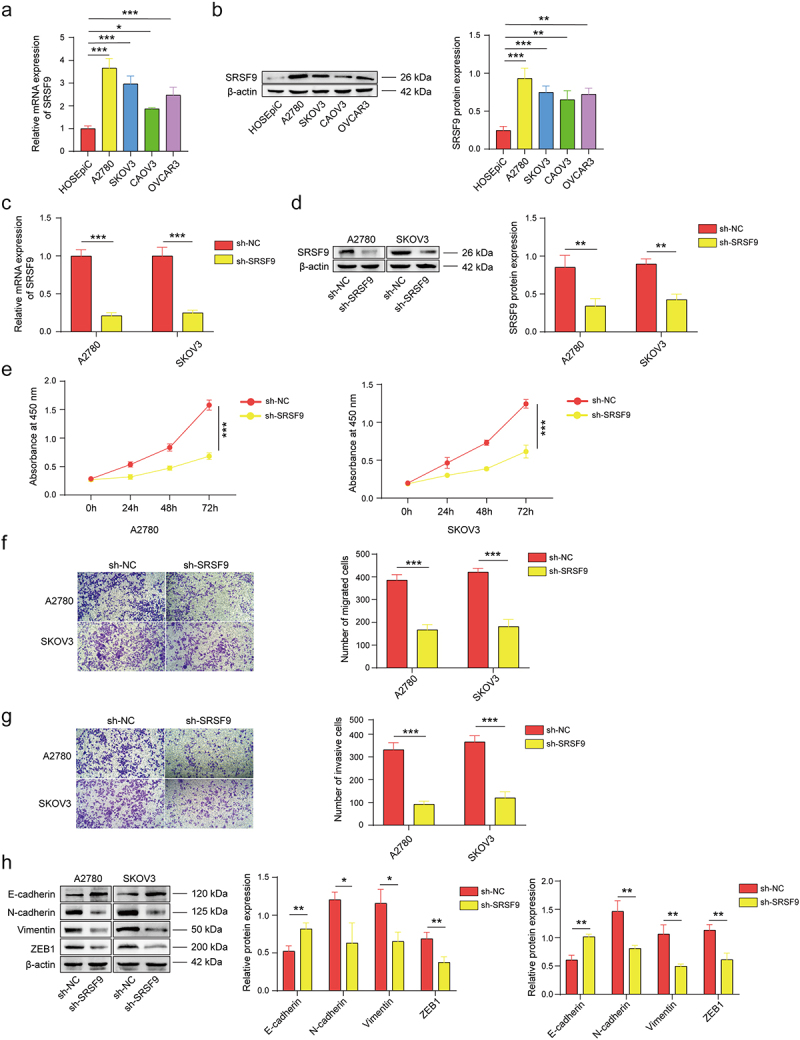
SRSF9 regulates cell proliferation, migration, invasion, and EMT in OC.

**Figure 2.**
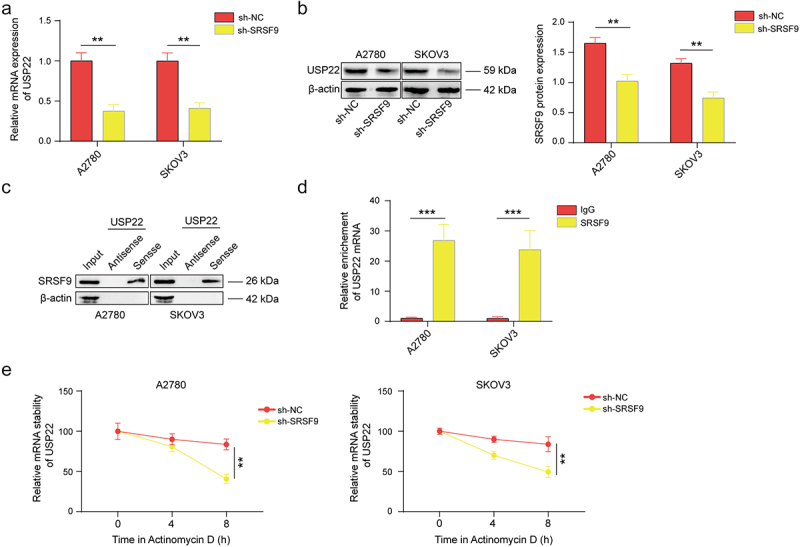
SRSF9 binds to USP22 mRNA and increases its expression.

We detected the expression of SRSF9 in OC cells and normal ovarian epithelial cells by western blotting and qPCR, and the results revealed that SRSF9 was overexpressed in OC cells, with the highest levels in A2780 and SKOV3 cells (Figure 1a,b). Thus, these two cell lines were employed for the following experiments. Then, we transfected OC cells with sh-SRSF9 or sh-NC and verified the transfection efficiency. As shown in Figure 2c,d, sh-SRSF9 markedly reduced SRSF9 expression at both the mRNA and protein levels. The results of the CCK-8 assay suggested that, compared with sh-NC, knockdown of SRSF9 effectively reduced the absorbance at 450 nm, indicating that cell proliferation was inhibited (Figure 1e). Through Transwell assays, we found that SRSF9 knockdown significantly suppressed the migration and invasion of OC cells (Figure 1f,g). The expression of EMT biomarkers (E-cadherin, N-cadherin, and Vimentin) and ZEB1 was also detected via western blotting, and the results revealed that knockdown of SRSF9 increased E-cadherin and decreased the expression of N-cadherin, Vimentin, and ZEB1 (Figure 1h). These results indicate that SRSF9 inhibition can alleviate the malignant characteristics of OC cells.

(a) SRSF9 mRNA expression in OC cell lines was detected via qPCR. (b) The protein levels of SRSF9 in OC cell lines were detected via western blotting. A2780 and SKOV3 cells were transfected with sh-NC or sh-SRSF9 vectors. (c-d) SRSF9 expression after transfection was monitored by qPCR and western blotting. (e) Cell proliferation was determined by CCK-8 assays. (f-g) Cell migration and invasion were monitored via Transwell assays. (h) The protein levels of E-cadherin, N-cadherin, Vimentin, and ZEB1 were evaluated via western blotting. All of the data are shown as the mean ± SD. *n* = 3. * *p* < .05, ** *p* < .01, *** *p* < .001.

(a-b) The impact of SRSF9 knockdown on USP22 mRNA and protein levels was detected by qPCR and western blotting in A2780 and SKOV3 cells transfected with sh-NC or sh-SRSF9 vectors. (c-d) The interaction between USP22 mRNA and SRSF9 was validated via RNA pull-down and RIP assays. (e) USP22 mRNA stability was detected by qPCR in cells transfected with sh-NC or sh-SRSF9. All of the data are shown as the mean ± SD. *n* = 3. ** *p* < .01, *** *p* < .001.

### SRSF9 binds to USP22 mRNA to increase its expression

Through bioinformatic analysis via the ENCORI website, we predicted that SRSF9 could bind to USP22 mRNA. To verify this prediction, we evaluated the mRNA and protein expression of USP22 after SRSF9 knockdown. The USP22 level was significantly downregulated, as shown in Figure 2a,b. Moreover, an RNA pull-down assay was performed, and the results indicated that SRSF9 was obviously pulled down by USP22 mRNA but not by antisense USP22 mRNA (Figure 2c). The RIP results also demonstrated that USP22 mRNA was abundantly present in the complex precipitated by the anti-SRSF9 antibody (Figure 2d). Furthermore, knockdown of SRSF9 reduced the stability of USP22 mRNA, as evidenced by the results of the actinomycin D assay (Figure 2e). These results suggest that SRSF9 binds to USP22 mRNA to improve its stability.

### USP22 is involved in the SRSF9-mediated malignant progression of OC cells

**Figure 3.**
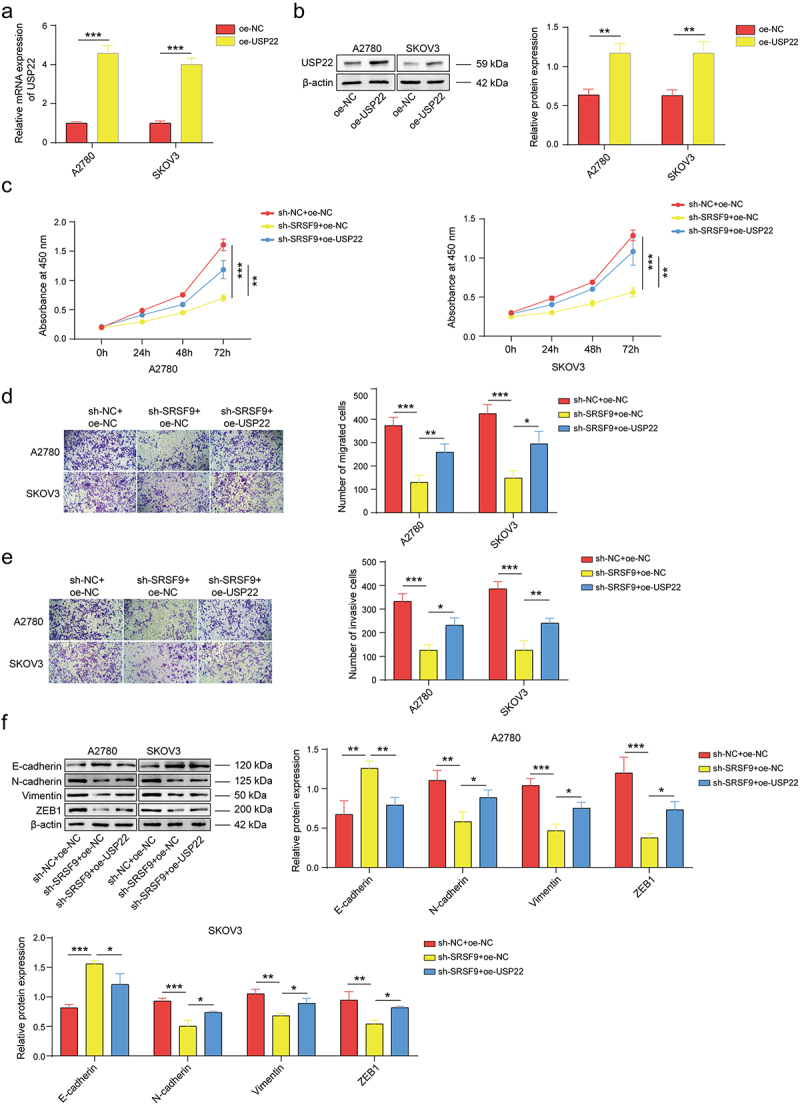
USP22 is involved in the SRSF9-mediated malignant progression of OC cells.

To reveal the role of USP22 in the protumor activity of SRSF9, we upregulated USP22 in OC cells. As shown in Figure 3a,b, the oe-USP22 vector significantly increased USP22 expression at both the mRNA and protein levels. Next, we detected the impact of USP22 upregulation on malignant phenotypes. The results of the CCK-8 assay suggested that USP22 upregulation inhibited the reduction in cell proliferation caused by SRSF9 knockdown (Figure 3c), and the Transwell results indicated that USP22 upregulation suppressed the decreased migration (Figure 3d) and invasion (Figure 3e) of OC cells caused by SRSF9 knockdown. Furthermore, the overexpression of USP22 inhibited the effect of SRSF9 suppression on the EMT of OC cells (Figure 3f). These results indicate that USP22 is involved in SRSF9-mediated cancer-promoting activities in OC cells.

(a-b) The expression of USP22 in A2780 and SKOV3 cells transfected with oe-NC or oe-USP22 vectors was monitored by qPCR and western blotting. A2780 and SKOV3 cells were transfected with sh-SRSF9 and/or oe-USP22. (c) Cell proliferation was determined by CCK-8 assays. (d-e) Cell migration and invasion were monitored via Transwell assays. (f) The protein levels of E-cadherin, N-cadherin, Vimentin, and ZEB1 were evaluated via western blotting. All of the data are shown as the mean ± SD. *n* = 3. * *p* < .05, ** *p* < .01, *** *p* < .001.

### USP22 deubiquitinates ZEB1 to increase its expression

**Figure 4.**
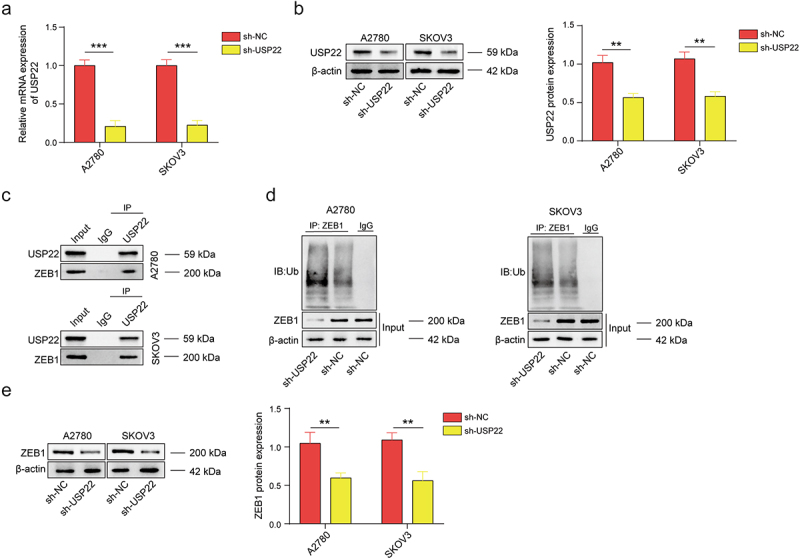
USP22 deubiquitinates ZEB1 and increases its expression.

USP22 can regulate ZEB1 expression, which may be associated with USP22-mediated deubiquitination.^18^ Here, we knocked down USP22 and detected the effects of USP22 on ZEB1 ubiquitination and ZEB1 expression. First, qPCR and western blot assays verified that sh-USP22 effectively suppressed USP22 expression (Figure 4a,b). Next, we employed a Co-IP assay and found that USP22 interacts with ZEB1 (Figure 4c). Moreover, USP22 knockdown markedly increased the level of ubiquitinated ZEB1 and reduced its expression level (Figure 4d,e). These results suggest that USP22 deubiquitinates ZEB1 to promote its expression.

(a-b) The mRNA and protein levels of USP22 in A2780 and SKOV3 cells transfected with sh-NC or sh-USP22 were detected by qPCR and western blotting. (c) The binding of USP22 with ZEB1 was validated by Co-IP. (d) The level of ubiquitinated ZEB1 in A2780 and SKOV3 cells transfected with sh-NC or sh-USP22 was detected via IP. (e) ZEB1 protein levels in A2780 and SKOV3 cells transfected with sh-NC or sh-USP22 were detected by western blotting. All of the data are shown as the mean ± SD. *n* = 3. ** *p* < .01, *** *p* < .001.

### USP22 promotes the malignant characteristics of OC cells by upregulating ZEB1

**Figure 5.**
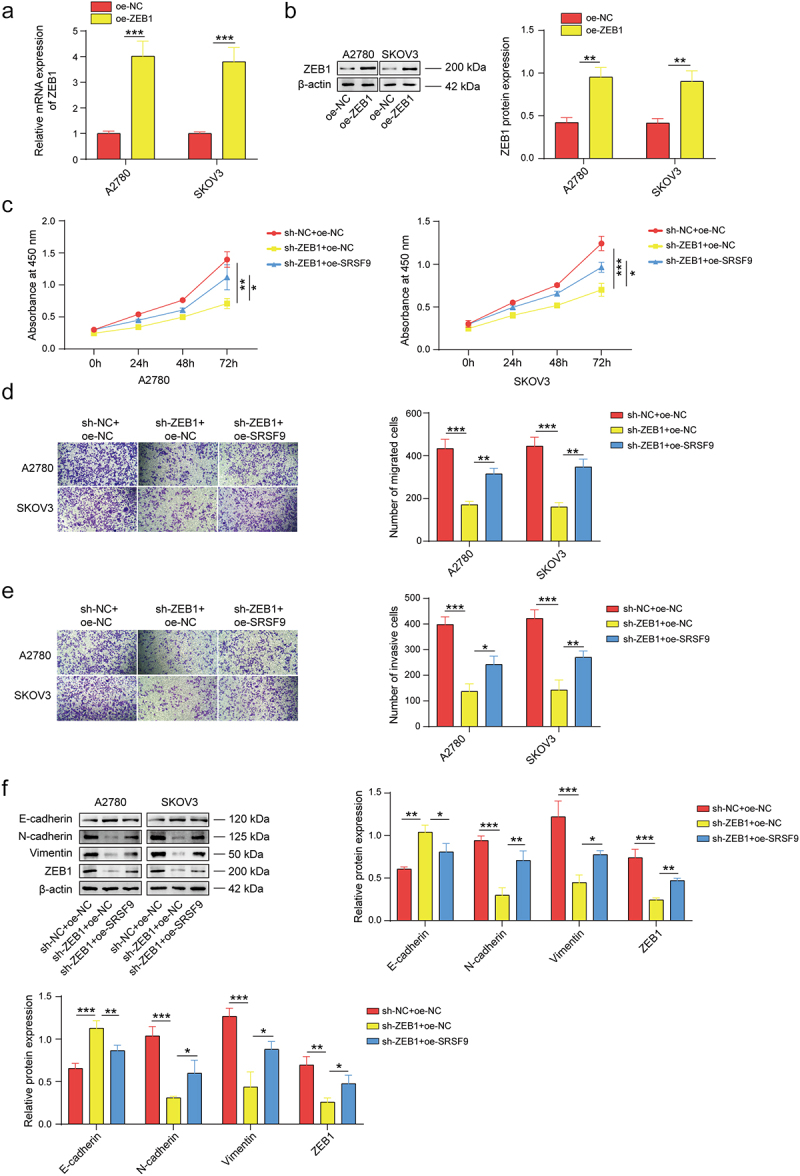
USP22 promotes the malignant characteristics of OC cells by upregulating ZEB1.

To verify the mechanism of the protumor activity of USP22, the oe-ZEB1 vector was introduced to upregulate ZEB1. As shown in Figure 5a,b, oe-ZEB1 transfection significantly elevated ZEB1 expression. Knockdown of USP22 markedly suppressed the proliferation of OC cells, but this effect was partially reversed by ZEB1 upregulation (Figure 5c). Additionally, USP22 knockdown significantly suppressed the migration and invasion of OC cells, while oe-ZEB1 transfection partially reversed this phenomenon (Figure 5d,e). Additionally, the reduction in USP22 led to a decrease in the levels of N-cadherin, Vimentin, and ZEB1 while increasing the level of E-cadherin. Conversely, the increase in ZEB1 partially reversed these changes (Figure 5f). Taken together, these findings indicate that USP22 promotes the proliferation, migration, invasion, and EMT of OC cells by upregulating ZEB1.

(a-b) ZEB1 expression in A2780 and SKOV3 cells transfected with oe-NC or oe-ZEB1 plasmids was monitored by qPCR and western blotting. A2780 and SKOV3 cells were treated with sh-USP22 and/or oe-ZEB1. (c) Cell proliferation was determined by CCK-8 assays. (d-e) Cell migration and invasion were monitored via Transwell assays. (f) The protein levels of E-cadherin, N-cadherin, Vimentin, and ZEB1 were evaluated via western blotting. All of the data are shown as the mean ± SD. *n* = 3. * *p* < .05, ** *p* < .01, *** *p* < .001.

### ZEB1 activates the transcription of SRSF9 and enhances the malignant progression of OC cells

**Figure 6.**
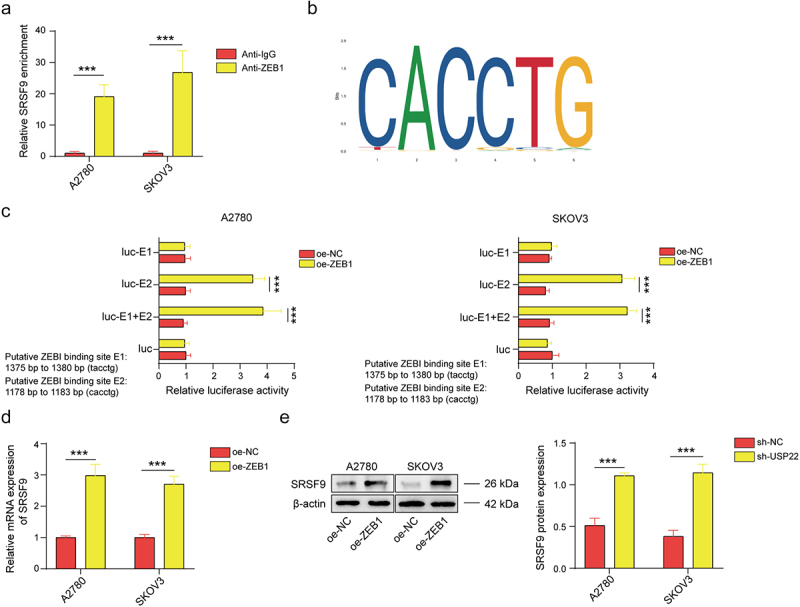
ZEB1 activates the transcription of SRSF9.

Through bioinformatics analysis using JASPAR software, we identified a potential interaction between ZEB1 and SRSF9. To verify this prediction, we employed a ChIP assay and found that ZEB1 could bind to the promoter sequence of SRSF9 (Figure 6a). We then employed two high-score loci according to JAPAR for further verification. As shown in Figure 6b,c, overexpression of the ZEB1 plasmid containing the E2 site significantly increased the luciferase activity, indicating that E2 is a functional binding site. Moreover, we detected SRSF9 expression after ZEB1 upregulation and found that SRSF9 expression was elevated at both the mRNA and protein levels (Figure 6d,e). Moreover, ZEB1 knockdown suppressed the proliferation, migration, and EMT of OC cells, but these effects were reversed by SRSF9 upregulation (Figure S1). These results demonstrate that ZEB1 activates the transcription of SRSF9 and enhances the EMT of OC cells.

(a) ZEB1 binding to the SRSF9 promoter was validated via a ChIP assay. (b) The ZEB1 binding motif was identified via the JASPAR database. (c) The binding site of ZEB1 with the SRSF9 promoter was validated by a luciferase assay. (d-e) The mRNA and protein levels of SRSF9 were evaluated by western blotting in A2780 and SKOV3 cells transfected with oe-NC or oe-ZEB1 plasmids. All of the data are shown as the mean ± SD. *n* = 3. *** *p* < .001.

### The SRSF9/USP22/ZEB1 positive feedback loop mediates OC tumorigenesis

**Figure 7.**
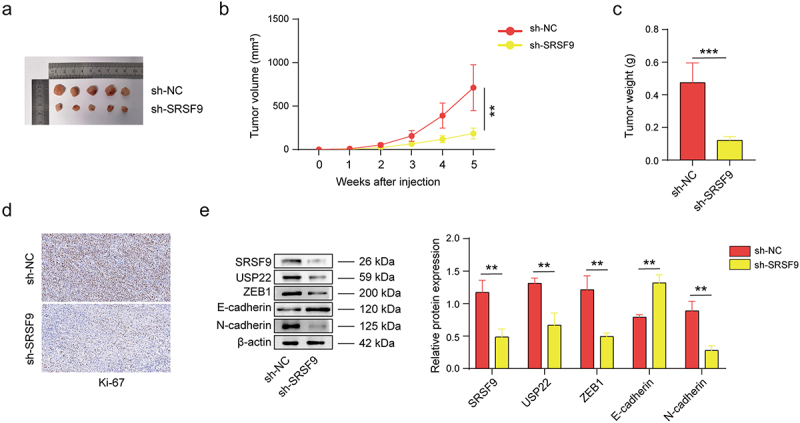
The SRSF9/USP22/ZEB1 positive feedback loop mediates OC tumorigenesis.

To verify the impact of SRSF9 on tumorigenicity *in vivo*, A2780 cells transfected with sh-SRSF9 or sh-NC were injected subcutaneously into nude mice. As shown in Figure 7a,c, compared with sh-NC transfection, sh-SRSF9 transfection significantly suppressed tumor growth and reduced tumor weight. Ki-67 expression was also reduced in the sh-SRSF9 group, as detected by IHC (Figure 7d). The western blotting results suggested that SRSF9 silencing decreased ZEB1, USP22, and N-cadherin expression levels but increased E-cadherin expression levels in tumor tissues (Figure 7e). Overall, the SRSF9/USP22/ZEB1 positive feedback loop mediates OC tumorigenesis.

(a-c) Mice (*n* = 5 per group) were subcutaneously injected with A2780 cells transfected with sh-NC or sh-SRSF9 vectors for 5 weeks, and representative images of xenograft tumors (a), growth curves of tumors (b), and average weight of the tumor mass (c) are presented. (d) Ki-67 expression was detected via IHC. (e) The protein levels of SRSF9, USP22, ZEB1, E-cadherin, and N-cadherin were detected via western blotting. All of the data are shown as the mean ± SD. ** *p* < .01, *** *p* < .001.

## Discussion

Approximately 80% of OC cases are initially diagnosed at advanced stages and are characterized by extensive metastases in the abdomen and pelvis. Despite treatments involving surgery and platinum/paclitaxel therapy, many patients develop drug resistance, leading to disease progression and poor prognosis.^20^ Hence, exploring novel biomarkers and treatment targets is imperative for improving patient prognosis. In our study, we investigated the protumor effects of SRSF9 in OC. We found that SRSF9 enhances proliferation and tumorigenicity *in vitro* and *in vivo* and that SRSF9 promotes invasion, migration, and EMT in OC cells. The identification of this positive feedback loop involving SRSF9, USP22, and ZEB1 offers new insights into the molecular mechanisms driving ovarian cancer progression. These findings raise the possibility of targeting SRSF9 as a novel therapeutic strategy against OC.

Posttranscriptional mechanisms play pivotal roles in determining the destiny of every transcript, including pre-mRNA processing, such as splicing and maturation; mRNA transport; and mRNA stability and translation.^21^ While these gene regulation processes are indispensable for normal cellular metabolism, their disruption can lead to various diseases, including cancer.^22,23^ RBPs exert significant control across RNA life cycle stages, influencing RNA abundance, structure, and function. Previous studies have shown elevated expression of SRSF9 in various cancers, such as bladder cancer, hepatocellular carcinoma, and OC.^24^ Consequently, RBPs have diverse versatile functions across different cancer types, contributing significantly to cancer initiation, tumor metabolism, and resistance to therapeutic agents.^25^ Previous studies have revealed that SRSF9 expression is increased in a range of tumor types, such as bladder cancer,^26^ hepatocellular carcinoma,^27^ and OC.^13^ Moreover, the downregulation of SRSF9 in cancer cell lines has been shown to impede malignant behaviors and trigger apoptosis.^26,27^ These results provide compelling evidence supporting the notion that SRSF9 functions as an oncogene. In our study, we found that the knockdown of SRSF9 significantly suppressed the malignant phenotypes of OC cells. Furthermore, we demonstrated that SRSF9 can bind to USP22 mRNA and improve its stability. The overexpression of USP22 partially rescued the influence of SRSF9 silencing on the malignant phenotypes of OC cells, suggesting that USP22 is involved in the SRSF9-mediated malignant progression of OC cells.

USP22 is an enzyme involved in histone modification, primarily by removing the monoubiquitin group from lysine 120 of H2B (H2Bub1).^28,29^ Increased USP22 has been correlated with worse outcomes in several cancers, such as pancreatic cancer,^30^ lung adenocarcinoma,^31^ and OC.^19^ Knockdown of USP22 in OC cells inhibits cell proliferation *in vitro* and tumor growth *in vivo*, indicating that USP22 is a potential therapeutic target for treating OC.^19^ Consistently, in this study, depletion of USP22 repressed cell proliferation, migration, invasion, and EMT in OC. In addition, USP22 has been reported to regulate ZEB1 ubiquitination to promote the transcription of downstream genes in hepatocellular carcinoma,^18^ and we considered whether a similar phenomenon could be observed in OC. Through a co-IP assay, we found that USP22 can bind to ZEB1 mRNA and that knockdown of USP22 markedly facilitates the ubiquitination of ZEB1 and thus enhances its protein degradation. The upregulation of ZEB1 also partially reversed the impact of USP22 silencing on the phenotypes of OC cells, indicating that USP22 promotes tumor progression by upregulating ZEB1.

EMT is a crucial biological process that plays a fundamental role in tumor metastasis.^32^ In response to various pathogenic stimuli, EMT drives the progression from early- to late-stage metastasis by increasing the adaptability and plasticity of cancer cells.^33^ ZEB1, a pivotal protein involved in EMT and critical for various biological processes, is distinguished by the presence of two zinc finger clusters that enable it to preferentially bind to DNA sequences known as E-boxes.^34^ Its typical role as a transcriptional regulator involves inhibiting or enhancing target gene expression by interacting with E-box motifs located in promoter regions. ZEB1 promotes tumorigenesis and metastasis in hepatocellular carcinoma by activating target gene transcription.^35^ In addition, a LINC00511/miR-524-5p/YB1/ZEB1 positive feedback mechanism enhances glioblastoma cell proliferation, EMT, and invasion.^36^ In OC, ZEB1 activates the transcription of glucose transporter 3 by interacting with its promoter region, facilitating cancer progression and metastasis.^15^ Therefore, we predicted the transcription factors of SRSF9 via the JASPAR website, and interestingly, we found that ZEB1 is a potential transcription factor of SRSF9. To verify this finding, we employed a ChIP assay and determined that ZEB1 can bind to the promoter sequence of SRSF9. A luciferase reporter assay also suggested that ZEB1 enhances SRSF9 promoter activity. Additionally, ZEB1 obviously increased SRSF9 levels. These findings suggest that ZEB1 promotes the transcription of SRSF9.

There are several limitations of this study. First, the study focused primarily on *in vitro* and xenograft animal models, and the expression levels of SRSF9, USP22, and ZEB1 in OC patients still needs to be determined. Additionally, this study predominantly examined the interactions among SRSF9, USP22, and ZEB1, and further research is needed to elucidate the broader regulatory networks and potential crosstalk with other signaling pathways in OC. Future studies should address these limitations by incorporating more comprehensive experimental models and exploring the interplay of SRSF9, USP22, and ZEB1 within a broader context.

## Conclusions

SRSF9 promotes the malignant phenotypes of OC through an SRSF9/USP22/ZEB1 positive feedback loop. The SRSF9/USP22/ZEB1 circuit may play a crucial role in OC progression and may be an important target for the diagnosis, treatment, and prognosis of OC. Nevertheless, this mechanism still needs further validation at the clinical level.

## Supplementary Material

Supplemental Material

## Data Availability

The datasets used or analyzed during the current study are available from the corresponding author upon reasonable request.

## Highlights

- SRSF9 enhances malignant phenotypes in ovarian cancer.
- SRSF9 enhances the stability of USP22 mRNA, and USP22 deubiquitinates ZEB1.
- ZEB1 upregulates SRSF9 expression through transcriptional activation, thus establishing a positive feedback loop.
- SRSF9 promotes the progression of ovarian cancer via a positive feedback circuit through the USP22/ZEB1 axis.
