# Correction

**DOI:** 10.1080/15384047.2024.2437847

**Published:** 2024-12-05

**Article title**: A positive feedback loop of SRSF9/USP22/ZEB1 promotes the progression of ovarian cancer

**Authors**: Wang, J., Hu, M., Min, J. & Li, X.

**Journal**: *Cancer Biology & Therapy*

**DOI**: https://doi.org/10.1080/15384047.2024.2427415

The author has been informed of issues with the group names and vertical coordinate labeling errors in Figures 2, 5 and 6.

**Figure 2.**
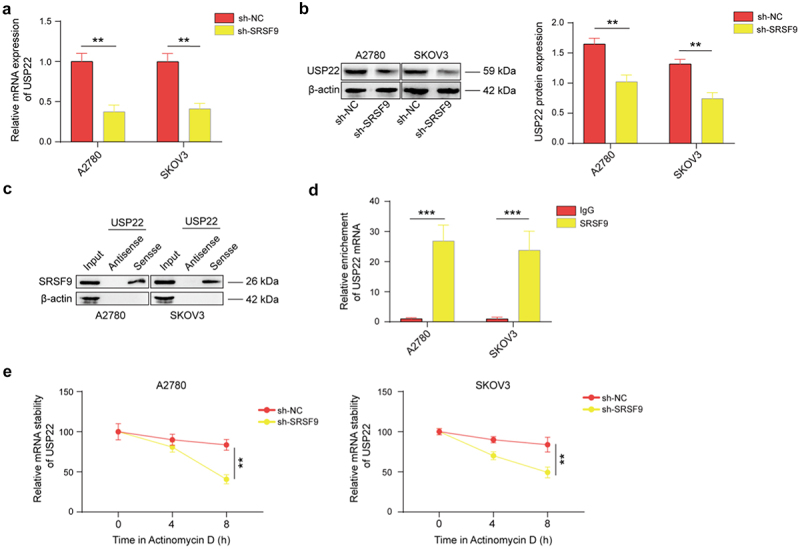

**Figure 5.**
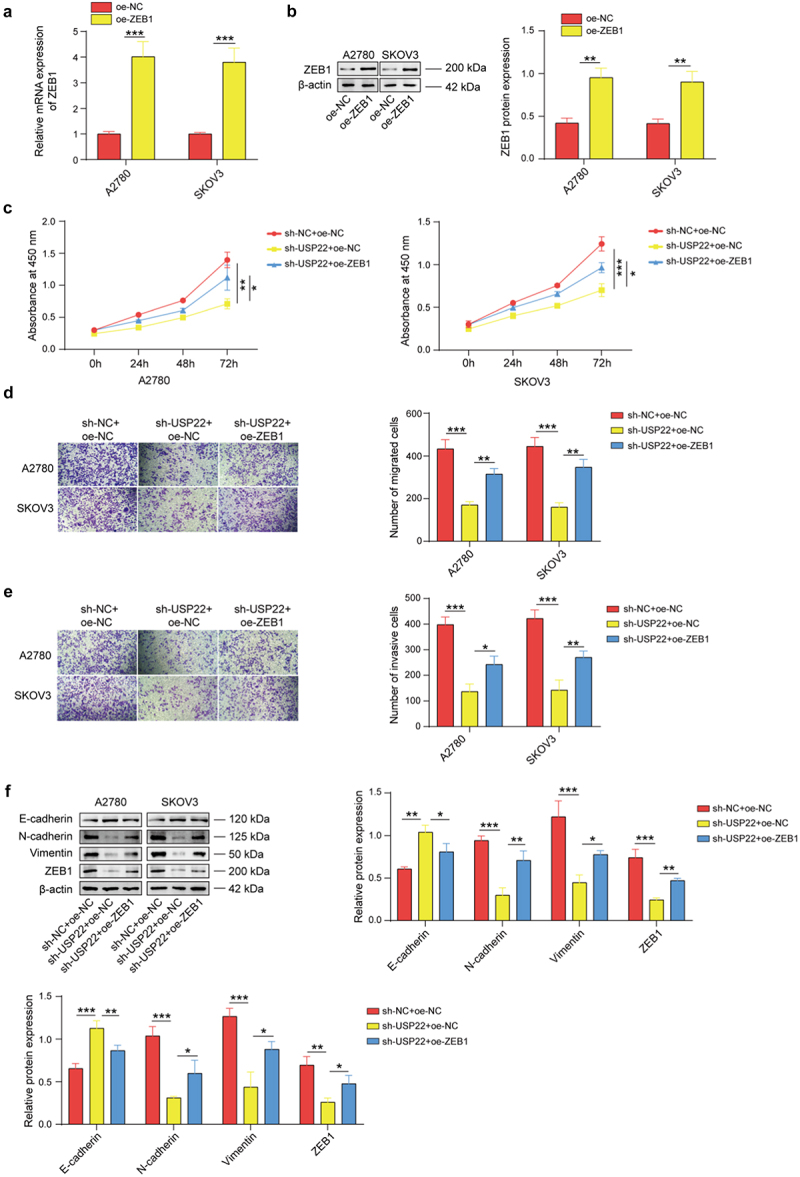

**Figure 6.**
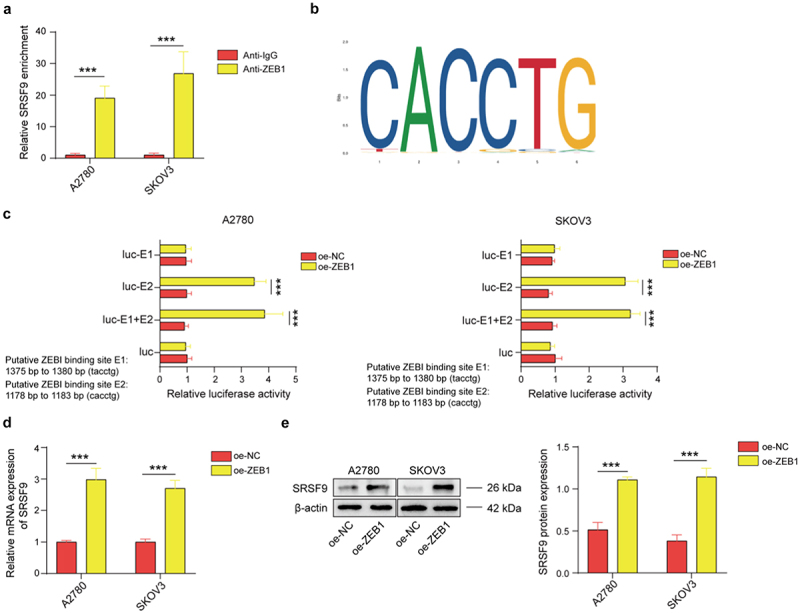

The author has requested that the current versions of Figures 2, 5 and 6 be replaced with the updated versions provided below, as they more accurately reflect the original intentions. The authors would like to thank the journal for the opportunity to clarify and correct this point and apologize for any inconvenience caused.

