# Supplementary material for: A positive feedback loop of SRSF9/USP22/ZEB1 promotes the progression of ovarian cancer
Source: Cancer Biol Ther. 2024 Nov 12;25(1):2427415. doi: 10.1080/15384047.2024.2427415 (PMC11559372; doi:10.1080/15384047.2024.2427415)
Supplement: Supplemental Material [file KCBT_A_2427415_SM6281.docx]

**Supplemental figure legends**

**Figure S1. ZEB1 promotes the malignant characteristics of OC cells by upregulating SRSF9.**

(A) ZEB1 expression in A2780 and SKOV3 cells transfected with sh-NC or sh-ZEB1 plasmids and SRSF9 expression in cells transfected with oe-NC or oe-ZEB1 were detected using qPCR. A2780 and SKOV3 cells were transfected with sh-ZEB1 and/or oe-SRSF9. (B) Cell proliferation was determined by CCK-8 assays. (C) Cell invasion was measured via Transwell assays. (D) The protein levels of E-cadherin and N-cadherin were evaluated by western blotting. All of the data are shown as the mean ± SD. n = 3. * p <0.05, ** p <0.01, *** p <0.001.
